# Positive nuclear BAP1 immunostaining helps differentiate non-small cell lung carcinomas from malignant mesothelioma

**DOI:** 10.18632/oncotarget.10653

**Published:** 2016-07-18

**Authors:** Michele Carbone, David Shimizu, Andrea Napolitano, Mika Tanji, Harvey I. Pass, Haining Yang, Sandra Pastorino

**Affiliations:** ^1^ Thoracic Oncology Program, University of Hawaii Cancer Center, Honolulu, HI, USA; ^2^ Department of Pathology, Queen Medical Center, Honolulu, HI, USA; ^3^ Department of Cardiothoracic Surgery, New York University, NYU Langone Medical Center, NY, USA

**Keywords:** mesothelioma, lung cancer, BAP1, differential diagnosis, immunohistochemistry

## Abstract

The differential diagnosis between pleural malignant mesothelioma (MM) and lung cancer is often challenging. Immunohistochemical (IHC) stains used to distinguish these malignancies include markers that are most often positive in MM and less frequently positive in carcinomas, and vice versa. However, in about 10–20% of the cases, the IHC results can be confusing and inconclusive, and novel markers are sought to increase the diagnostic accuracy.

We stained 45 non-small cell lung cancer samples (32 adenocarcinomas and 13 squamous cell carcinomas) with a monoclonal antibody for BRCA1-associated protein 1 (BAP1) and also with an IHC panel we routinely use to help differentiate MM from carcinomas, which include, calretinin, Wilms Tumor 1, cytokeratin 5, podoplanin D2-40, pankeratin CAM5.2, thyroid transcription factor 1, Napsin-A, and p63. Nuclear BAP1 expression was also analyzed in 35 MM biopsies. All 45 non-small cell lung cancer biopsies stained positive for nuclear BAP1, whereas 22/35 (63%) MM biopsies lacked nuclear BAP1 staining, consistent with previous data. Lack of BAP1 nuclear staining was associated with MM (two-tailed Fisher's Exact Test, *P* = 5.4 × 10^−11^). Focal BAP1 staining was observed in a subset of samples, suggesting polyclonality. Diagnostic accuracy of other classical IHC markers was in agreement with previous studies. Our study indicated that absence of nuclear BAP1 stain helps differentiate MM from lung carcinomas. We suggest that BAP1 staining should be added to the IHC panel that is currently used to distinguish these malignancies.

## INTRODUCTION

The incidence of malignant mesothelioma (MM) has increased exponentially in the US since the early ‘60s, reaching 3,200 cases per year at the beginning of this century, and has remained stable since then [1]. Similarly, the incidence of lung cancer has increased exponentially during the past century, with currently over 200,000 cases of lung cancer diagnosed per year in the US [2]. Comparable trends have been observed since World War II in most countries, as a consequence of the increased use of asbestos, the most common cause of MM, and of cigarette smoking, the most common cause of lung cancer [3]. Moreover, asbestos and smoking synergize in causing lung cancer, and co-factors may increase asbestos carcinogenicity and MM [4–6]. Also, exposure to asbestos and other carcinogenic fibers present in the environment can cause MM and probably lung cancer [7–9].

MM and lung cancer patients are treated differently and have different prognosis, thus it is very important to properly diagnose these malignancies. This differential diagnosis is difficult, because MMs, in particular the epithelial subtype –which comprises about 70% of all MMs– can show a morphology similar to that of non-small cell lung carcinomas, and lung carcinosarcomas and spindle cell carcinomas can have a morphology similar to biphasic and sarcomatoid MMs. A set of immunohistochemical (IHC) stains helps distinguish these malignancies [10, 11]: more than 80% of epithelial MMs show nuclear stain for Wilms tumor protein (WT1) and calretinin, and show membranous stain for cytokeratin 5 (CK5). Lung squamous cell carcinomas (SCC) stain positive for CK5, and also for p63 (nuclear), and p40 (nuclear), the latter two markers are negative in MMs. Moreover, about 40% of SCC can also be positive for calretinin and show membranous stain for podoplanin (D2-40). Lung adenocarcinomas are instead positive for nuclear thyroid transcription factor 1 (TTF-1) and Napsin-A and for other cytoplasmic epithelial markers, such as the carcinoembryonic antigen (CEA), the Epithelial Related Antigen (MOC31), EpCam (BEREP4), etc., and rarely for calretinin and podoplanin (D2-40). Pankeratin CAM5.2 stains the membranes and the cytoplasm of the cells in both MMs and lung carcinomas.

When the results of the IHC stains fit the expectations, the diagnosis is usually straightforward. In about 10–20% of the cases, however, these malignancies can produce conflicting IHC results, with both MM and lung carcinoma markers being either positive or negative in the same tumor, or showing only a fraction of tumor cells being positive. Accordingly, there are still a large number of MMs that are misdiagnosed. In a large follow-up study that covered 25% of the French population, Goldberg *et al*. reported that the initial diagnosis of MM was confirmed only in 67% of cases [12]. Most recently, a review of Chinese MM confirmed the initial diagnosis, which was made with the support of a panel of IHC stains, in only 56% of cases [9]. In our experience, 10% or more of MMs, in the US, are misdiagnosed: these cases are often from hospitals and pathologists that rarely see patients with these types of tumors. Thus, too many patients worldwide continue to be misdiagnosed and consequently do not receive proper treatment for their malignancy.

Following studies of an epidemic of MM in Cappadocia that we linked to gene-environment interaction [13, 14], we discovered that germline truncating mutations in the BRCA1 associated protein-1 (*BAP1*) gene caused a very high incidence of MM, in some families in the US and abroad, in the absence of occupational exposure to asbestos [15, 16]. Moreover, using a BAP1^+/−^ mouse model, we demonstrated that mice, carrying germline *BAP1* mutations, develop MM following exposure to very low doses of asbestos that rarely caused MM in wild-type mice [17]. Our data, confirmed and expanded by others, showed that germline *BAP1* mutations are also associated with uveal melanoma, renal cell carcinoma and other malignancies, causing a condition that we named “BAP1 cancer syndrome” [18]. BAP1 is a member of the ubiquitin C-terminal hydrolase subfamily of deubiquitinating enzymes and is found associated with multi-protein complexes that regulate cell cycle, differentiation, apoptosis, gluconeogenesis, and the DNA damage response [18, 19].

Somatic *BAP1* mutations were also detected in sporadic (i.e., non familiar) MM [15, 20–22]. Using multidimensional genetic analyses, and IHC we demonstrated BAP1 inactivation in >60% of sporadic MMs [23], making *BAP1* the most commonly mutated gene in MM, a finding confirmed by others [24–26]. These findings underscore the pivotal role of BAP1 in MM. Recently, several studies reported that lack of nuclear BAP1 immunostaining helps differentiating benign reactive pleural effusion and pleurisy, which are BAP1 positive, from MMs, which are frequently BAP1 negative [27–30]. Other malignancies instead express normal levels of BAP1: for example BAP1 is expressed and detected by IHC in most pancreatic carcinomas [31], and in most peritoneal and gynecologic serous adenocarcinomas [32]. In 2012, Fan *et al.* detected BAP1 by Western blot studies in 103 non-small cell lung cancers, and correlated high expression with a good prognosis [33].

Here, we tested the hypothesis that BAP1 immunostain might help improve the accuracy of the differential diagnosis between MM, which often shows no BAP1 nuclear staining, and lung cancer, which we predicted to be BAP1 positive.

## RESULTS AND DISCUSSION

**Figure 1:**
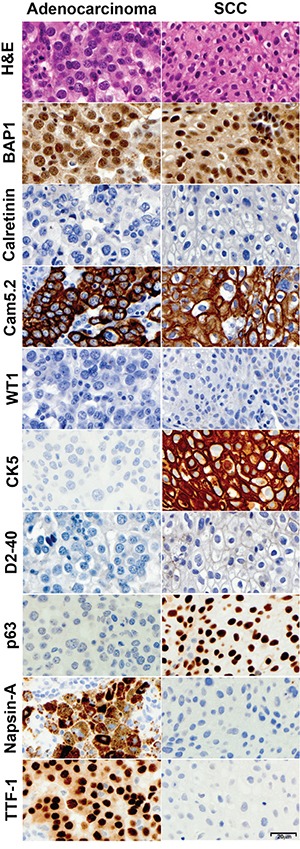
Immunohistochemical characterization of non-small cell lung cancers Representative lung adenocarcinoma (left) and SCC (right) were stained with Hematoxylin and Eosin, and for expression of BAP1, calretinin, CAM5.2, WT1, CK5, D2-40, p63, Napsin-A and TTF-1. Note the strong BAP1 nuclear staining in both specimens. All photomicrographs were taken at 400x original magnification; representative size bar is shown on the bottom right panel.

All 45 non-small cell lung cancer samples analyzed –32 adenocarcinomas and 13 SCC– stained positive for nuclear BAP1 (Table 1, Figure 1). Strong nuclear staining was detected in ~100% of the tumor cells in all these tumors, except for 2 adenocarcinomas, in which some tumor areas contained cells showing BAP1 nuclear staining and some areas contained tumor nodules that were BAP1 negative. These cases are possibly due to presence of tumor sub-clones that had lost BAP1 expression, underscoring the risk of possible sample error if only minute needle biopsies, or tumor-arrays (slides with multiple minute fragments of different tumors) were to be examined [34].

**Table 1:** Immunoreactivity of nuclear BAP1 in malignant mesothelioma and non-small cell lung cancer

Tumor Type	Malignant Mesothelioma				Non-small cell lung cancer		
Histology	Epithelial	Biphasic	Sarc	Total	Adeno	SCC	Total
**Sample no.**	20	8	7	35	32	13	45
**BAP1 Neg**	13 (65%)	4 (50%)	5 (71%)	22 (63%)	0	0	0
**BAP1 Pos**	1 (5%)	1 (13%)	2 (29%)	4 (11%)	30 (94%)	13 (100%)	43 (96%)
**BAP1 Focal**	6 (30%)	3 (37%)	0	9 (26%)	2 (6%)	0	2 (4%)

**Figure 2:**
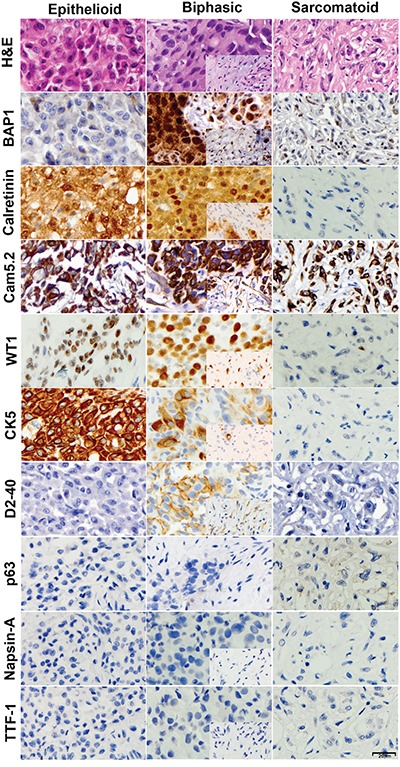
Immunohistochemical characterization of malignant mesotheliomas Representative epithelioid (left), biphasic (center) and sarcomatoid (right) specimens were stained with Hematoxylin and Eosin, and for expression of BAP1, calretinin, CAM5.2, WT1, CK5, D2-40, p63, Napsin-A and TTF-1. Inserts depict the spindle component within the biphasic tumor. Note lack of BAP1 nuclear staining in the epithelioid and sarcomatoid specimens. All photomicrographs were taken at 400x original magnification; representative size bar is shown on the bottom right panel.

In parallel, we stained 35 new MM samples for BAP1. Overall, BAP1 expression was entirely lost in 22/35 (63%) of all MM samples. (Table 1, Figure 2). Focal BAP1 staining, suggestive of polyclonality [35], was observed in 6/20 (30%) of epithelial MMs and 3/8 (37%) of biphasic MMs. These results are consistent with our previous study in which, using integrated genetic approaches, coupled with IHC, we found that 66% of 92 MM studied displayed a lack of nuclear BAP1 staining [23]. A re-analysis of these cases revealed that, among the 60/92 MMs that were of the epithelial-type, only 14 (23%) were BAP1 positive and 1 additional MM showed focal nuclear positivity. Among the 32/92 non-epithelial MMs (i.e., biphasic and sarcomatoid), 11 (30%) were BAP1 positive, and 5 (15%) showed focal positivity.

In addition, we compared a panel of “classical” IHC stains used to distinguish MM and lung cancer in these 45 non-small cell lung cancer samples and in 10 MM samples (Table 2). MM cells were mostly positive for WT1 (nuclear), calretinin (nuclear and cytoplasmic), D2-40, CK5, and CAM5.2, and the same cells were negative for TTF-1, p63, and Napsin-A. Lung adenocarcinoma cells were always positive for TTF-1, Napsin-A, and CAM5.2; and negative for nuclear WT1 and D2-40 and 6% of them stained for calretinin (nuclear and cytoplasmic focal staining). Tumor cells in SCC were almost uniformly positive for CK5, CAM5.2 and p63, and negative for WT1, TTF-1 and Napsin-A; Calretinin and D2-40 were focally positive respectively in 23% and 77% of them (Table 2, Figures 1-2). These findings are in agreement with previous studies [10, 11] indicating that WT1 nuclear positivity is the most specific positive marker for MM, while TTF-1 and Napsin-A are most specific for lung adenocarcinoma, and p63 and p40 are specific markers for lung SCC. We found that calretinin, a marker often used in support of the diagnosis of MM, is certainly a very sensitive MM marker, but because it stains also a large proportion of SCC and some adenocarcinomas it is insufficient, *per se*, to establish the diagnosis. It has been our experience that, at times, misdiagnoses of MM were based on an incomplete, limited, set of IHC stains showing positivity for calretinin. The more specific marker WT-1 stains about 80% of the epitelioid MM, and about 50% of sarcomatoid MMs. We found that D2-40 stains MM but also lung carcinomas and therefore, although sensitivity is high, as most MMs stain for D2-40, the specificity is low.

**Table 2:** Immunoreactivity of other markers in malignant mesothelioma and non-small cell lung cancer

Tumor Type	Malignant Mesothelioma				Non-small cell lung cancer		
Histology	Epithelial	Biphasic	Sarc	Total	Adeno	SCC	Total
**Sample no.**	5	1	4	10	32	13	45
**Calretinin**	5 (100%)	1 (100%)	3 (75%)	9 (90%)	2 (6%)	3^$^ (23%)	5 (11%)
**Cam5.2**	5 (100%)	1 (100%)	4 (100%)	10 (100%)	32 (100%)	13*** (100%)	45 (100%)
**D2-40**	4^#^ (100%)	1* (100%)	3$ (75%)	8 (80%)	0	10$ (77%)	10 (4%)
**WT1**	5 (100%)	1 (100%)	2 (50%)	8 (80%)	0	0	0
**CK 5**	5* (100%)	1* (100%)	3 (75%)	9 (90%)	2* (6%)	13 (100%)	15 (35%)
**p63**	0	0	0	0	4^$^ (12%)	13 (100%)	17 (38%)
**TTF-1**	0	0	0	0	31 (97%)	1** (8%)	32 (71%)
**Napsin-A**	0	0	0	0	31** (97%)	1 (8%)	32 (71%)

# for one sample D2-40 staining was not available; three out of the four positive MM samples showed focal staining.

* focal staining was observed in one positive sample.

** focal staining was observed in two positive samples.

*** focal staining was observed in three positive samples.

$ focal staining was observed in all the positive samples.

In summary, we found that lack of BAP1 nuclear staining was preferentially associated with MM (two-tailed Fisher's Exact Test, *P* = 5.4 × 10^−11^) and that instead lack of nuclear staining is not found in lung carcinomas, or at least is quite rare, since in our study 45/45 lung cancers stained for nuclear BAP1. In support of our findings, genomic data from the TCGA collaboration on lung cancer showed that mutations of *BAP1* are extremely rare in non-small cell lung cancer: frame-shift mutations and deletions that would result in loss of BAP1 nuclear staining were present in less than 1% of more than 400 lung adenocarcinomas [36–38] and 178 SCC studied [39]. Moreover, this June 2016, after our paper was submitted for publication, Andrici J et al., reported that out of 155 lung adenocarcinomas and 72 lung SCC, only one had lost BAP1 expression [40]. These Authors, quoting previous literature, noted: “this finding increases the specificity of loss of expression for BAP1 for the diagnosis of mesothelioma” [40]. Although the paper by Andrici et al. did not include a parallel analysis of MM biopsies, their IHC results independently support our findings and conclusions. Together, these findings, justify including BAP1 in the panel of antibodies used to differentiate lung cancer from MM.

## MATERIALS AND METHODS

All investigations described in this study have been performed in accordance with the principles embodied in the Declaration of Helsinki. Written informed consent was received from all patients. Collection and use of patient information and samples were approved by the IRB of the University of Hawaii (IRB no. 14406). We studied 32 primary lung adenocarcinomas, 13 primary lung SCC and 10 MM biopsies, which were diagnosed at the Queens Medical Center, Honolulu, Hawaii. We also analysed 25 MM biopsies from the New York University New York, New York, (all calretinin and WT1 positive and negative for epithelial markers) for a total of 35 MM biopsies. Of these, 20 were of the epithelial type, 8 were biphasic and 7 were sarcomatoid.

IHC was performed on formalin fixed paraffin embedded tissue sections, using the avidin-biotin-peroxidase complex method in a DAKO-autostainer (Carpinteria, CA, USA). The primary antibodies used in this study were: BAP1 (Clone C-4, Santa Cruz Biotechnology); p63 (clone 4A4, Biocare); Napsin A (clone BC15, Biocare); TTF1 (clone SPT24, Leica); CK5 (clone XM26, Leica); WT1 (clone WT49, Leica); Calretinin (clone CAL6, Leica); D2-40, clone D2-40, DAKO); Cam5.2 (clone Cam 5.2, BD Biosciences).

All diagnoses were made on hematoxylin-eosin stained sections combined with immunohistochemical and clinical features. Expert pathologists in pleural pathology, independently evaluated the biopsies (M.C., D.S and H.I.P.).

IHC of BAP1 protein expression was performed as described [15, 23, 41], using a mouse monoclonal anti-BAP1antibody (C-4: Santa Cruz Biotechnology, TX). This antibody recognizes the epitope between a.a. 430 and 739; therefore, it detects BAP1 wild-type and mutant forms that retain the nuclear localization signal (NLS). We extensively validated this antibody for the detection of nuclear BAP1 on a number of normal human pleural samples –all showing nuclear staining in 100% of pleural cells, as well as on MM-derived cell lines [41]. Statistical analysis was performed applying the two-tailed Fisher's Exact Test.
